# Label-Free Rapid Separation and Enrichment of Bone Marrow-Derived Mesenchymal Stem Cells from a Heterogeneous Cell Mixture Using a Dielectrophoresis Device

**DOI:** 10.3390/s18093007

**Published:** 2018-09-08

**Authors:** Junya Yoshioka, Yu Ohsugi, Toru Yoshitomi, Tomoyuki Yasukawa, Naoki Sasaki, Keitaro Yoshimoto

**Affiliations:** 1 Department of Life Sciences, Graduate School of Arts and Sciences, The University of Tokyo, Tokyo 153-8902, Japan; j_yoshioka@bio.c.u-tokyo.ac.jp (J.Y.); y-oosugi@g.ecc.u-tokyo.ac.jp (Y.O.); t_yoshitomi@bio.c.u-tokyo.ac.jp (T.Y.); 2 Department of Applied Chemistry, Faculty of Science and Engineering, Toyo University, Saitama 350-8585, Japan; nsasaki@toyo.jp; 3 Graduate School of Material Science, University of Hyogo, Hyogo 678-1297, Japan; yasu@sci.u-hyogo.ac.jp; 4 JST, PRESTO, The University of Tokyo, Tokyo 153-8902, Japan

**Keywords:** dielectrophoresis, stem cell, cell enrichment, label-free separation

## Abstract

Bone marrow-derived mesenchymal stem cells (BMSCs) are an important cell resource for stem cell-based therapy, which are generally isolated and enriched by the density-gradient method based on cell size and density after collection of tissue samples. Since this method has limitations with regards to purity and repeatability, development of alternative label-free methods for BMSC separation is desired. In the present study, rapid label-free separation and enrichment of BMSCs from a heterogeneous cell mixture with bone marrow-derived promyelocytes was successfully achieved using a dielectrophoresis (DEP) device comprising saw-shaped electrodes. Upon application of an electric field, HL-60 cells as models of promyelocytes aggregated and floated between the saw-shaped electrodes, while UE7T-13 cells as models of BMSCs were effectively captured on the tips of the saw-shaped electrodes. After washing out the HL-60 cells from the device selectively, the purity of the UE7T-13 cells was increased from 33% to 83.5% within 5 min. Although further experiments and optimization are required, these results show the potential of the DEP device as a label-free rapid cell isolation system yielding high purity for rare and precious cells such as BMSCs.

## 1. Introduction

Mesenchymal stem cells (MSCs), one type of somatic stem cells, possess a self-renewal property and the ability to differentiate into not only mesodermal lineages, such as chondrocytes, osteocytes, adipocytes [1,2,3], but also endodermal [4,5,6] and ectodermal lineages [3,7,8,9]. Since stem cell-based therapy has recently emerged as a promising regenerative medicine, the technology of cell separation has become more important. Bone marrow is the predominant MSC source and mainly contains non-adherent hematopoietic cells and adherent stromal cells, including bone marrow-derived MSCs (BMSCs). The fluorescence-activated cell sorting (FACS) method is currently used for cell separation [10,11]; however, it is time-consuming, and requires large equipment with high operating costs and cell labelling. In particular, long-term cell staining with antibodies may interfere with the clinical use of the cells after separation and is not suitable for cell samples containing blood coagulation factors [12]. Thus, the density-gradient method is generally used for cell isolation from the bone marrow, which is based on separation by cell size and density after the collection of tissue samples [13,14,15]. Although this method does not need cell labelling, it has limitations with regard to purity, repeatability, and long centrifugation time. For instance, the typical centrifugation time is about 40 min, and the purity of monocytes and dendritic cells from bone marrow after density-gradient separation was reported to be around 10% [15]. Therefore, development of alternative label-free cell separation systems for BMSCs with short separating time and high purity is desired in the field of stem cell research.

Dielectrophoresis (DEP) has attracted much attention as a manipulation technique for cells [16,17,18,19]. DEP is based on the interaction between a non-uniform electric field and the polarization charge on the surface of cells. The cell type, cell size, and composition of cytoplasm affect their DEP behavior. Depending on the degree of polarization of the cells relative to that of the suspending medium, two types of DEP forces are induced. In the case of positive DEP (p-DEP), the polarizability of cells is greater than that of the suspending medium and the cells migrate towards high electric field regions, resulting in cell capture on the electrodes. On the other hand, in the case of negative DEP (n-DEP), cells are less polarizable than the suspending medium and they move away from high electric field regions and float between the electrodes. This DEP behavior of cells has been utilized for separation of viable and non-viable cells [20,21], microalgae with different lipid contents [22], and cancer cells [23]. If separation of BMSCs is achieved by DEP-based methods, it potentially could become the dominant method instead of conventional separation methods. In the present study, rapid separation of unlabeled cells by DEP was conducted using two kinds of cells that are derived from bone marrow; the human mesenchymal stem cell line (UE7T-13) and the human promyelocytic leukemia cell line (HL-60) were used as the models of BMSCs and promyelocytes, respectively.

## 2. Materials and Methods

### 2.1. Fabrication of Electrodes and the Dielectrophoresis (DEP) Device

A fabrication method for a saw-shaped electrode on glass surface has been reported previously [24]. Briefly, a positive photoresist was coated by spin coater on an indium tin oxide (ITO) glass (Geomatec Co., Ltd., Yokohama, Japan), and UV light (254 nm, 0.32 mW/cm^2^) was irradiated through a saw-shaped photomask for 8.5 s. The thickness of the ITO electrode on the glass was 200 nm. The basal plate was immersed in an etchant solution ITO-02 (Kanto Chemical Co., Inc., Tokyo, Japan) under ultrasonic irradiation to remove any unnecessary part of the ITO electrode. Thirty-micrometer-thick polyester films (Nitto Denko Corp., Osaka, Japan) were used as a spacer in the DEP device and were sandwiched between the upper and lower substrates having a flat and saw-shaped ITO electrode, respectively. In this study, the electrode width was 20 μm.

### 2.2. Simulation Using the Finite Element Method (FEM)

The finite element method (FEM) was performed to simulate the distribution of the electric field strength on the saw-shaped electrodes in the DEP device, using the commercial simulation software package COMSOL Multiphysics (COMSOL, Inc., Burlington, MA, USA). All simulations were undertaken in three-dimensions and the potentials on the electrodes were set to 10 V.

### 2.3. Cell Culture

The UE7T-13 cell line and HL-60 cell line were purchased from the Japanese Collection of Research Bioresources (Tokyo, Japan) and RIKEN Bio-Resource Center (Tsukuba, Japan), respectively. UE7T-13 cells were cultured in Dulbecco’s modified Eagle’s medium (DMEM) (Wako Pure Chemical Industries, Ltd., Osaka, Japan) containing 10% fetal bovine serum, 0.05 mg/mL penicillin, 0.05 mg/mL streptomycin, and 0.1 mg/mL neomycin at 37 °C and 5% CO_2_. HL-60 cells were cultured in Roswell Park Memorial Institute medium-1640 (Wako Pure Chemical Industries, Ltd., Osaka, Japan) containing 10% fetal bovine serum, 0.05 mg/mL penicillin, 0.05 mg/mL streptomycin, and 0.1 mg/mL neomycin at 37 °C and 5% CO_2_.

### 2.4. Microscopic Observation of DEP Behaviors of the UE7T-13 and HL-60 Cells

Before cell injection into the DEP device, a blocking reagent N102 (NOF Corp., Tokyo, Japan) was coated onto the surface of the microfluidic channel in the device for 10 min. The UE7T-13 cells and HL-60 cells were suspended in 250 mM sucrose medium (conductivity: 1 mS/m) because sucrose medium not only makes the cells more polarizable than the surrounding medium, but also protects the cells from stress due to osmotic pressure. Both cells were stirred by pipette carefully to prevent cell aggregations before injection. After cell injection into the DEP device with micropipette manually, AC voltage was applied using Waveform Generator 33,500 B Series (Agilent Technologies International Japan, Ltd., Hachioji, Japan). The DEP behaviors of the UE7T-13 and HL-60 cells were observed under an optical microscope under conditions ranging from 30 kHz to 10 MHz with 20 V peak-to-peak (Vpp) AC voltage.

### 2.5. Viability Assay

The viability of UE7T-13 cells was investigated by live/dead assay, using Live-Dead Cell Staining Kit (BioVision Inc., Milpitas, CA, USA) after applying p-DEP with 7.5 Vpp at 100 kHz for 5 min and culturing for 2 h. Before cell culture, the sucrose solution was replaced by DMEM and the DEP device was immersed into DMEM. The UE7T-13 cells were incubated at 37 °C and 5% CO_2_. This assay was performed in triplicate.

### 2.6. Calculation of Purity, Recovery, and Enrichment Rate of UE7T-13 Cells after DEP Separation

The purity was calculated as the percentage of UE7T-13 cells in a total cell suspension. Recovery was calculated as the percentage of captured UE7T-13 cells per initial number of UE7T-13 cells. Enrichment was calculated as the ratio of the purity of UE7T-13 after DEP to that before DEP. All cell numbers were observed and counted by microscopy imaging. For separation and enrichment of the UE7T-13 cells, an AC voltage of 5.0 Vpp was applied at 30 kHz for 5 min. This experiment was performed in triplicate.

## 3. Results and Discussion

### 3.1. Electrode Design and Assessment of Cell DEP Response

**Figure 1:**
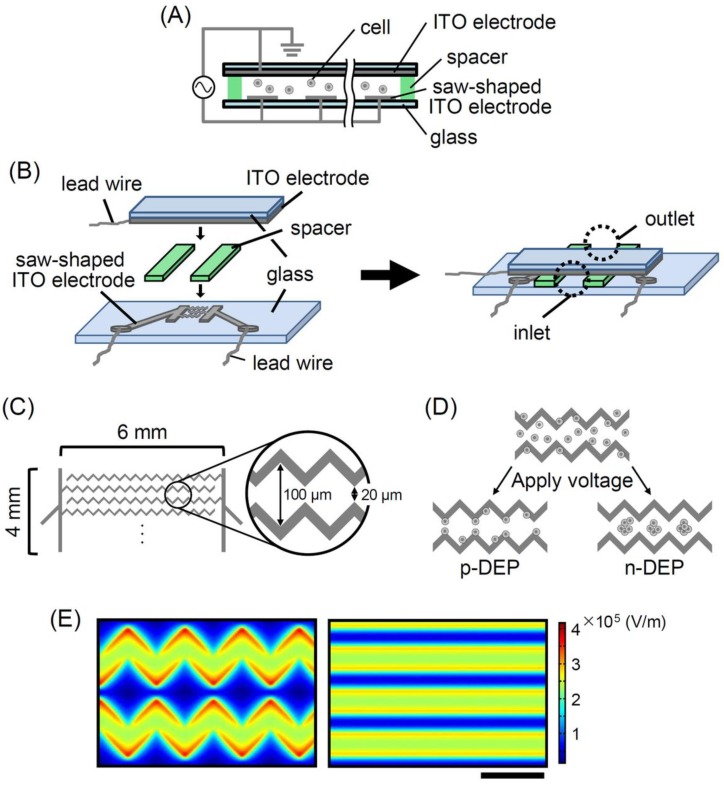
Structural overview of the dielectrophoresis (DEP) device. Schematic diagram of (**A**) cross-section and (**B**) bird’s eye view of the dismantled and constructed DEP devices. (**C**) Saw-shaped indium tin oxide (ITO) electrode printed on the bottom of the DEP device. (**D**) Illustration of p-DEP and n-DEP on the saw-shaped ITO electrode in the device. (**E**) Numerical simulation of electric field strength on (left) the saw-shaped ITO electrodes and (right) the line-shaped ITO electrodes by the finite element method (FEM). The potentials on the electrodes were set to 10 V. The height of calculated x-y plane is 4 μm from the bottom and scale bar represents 100 μm.

The cross-sectional view of the flow path in the device, the bird’s eye view of the dismantled and constructed devices, and top view of the electrode are shown in Figure 1A–C, respectively. The saw-shaped electrodes allow the cells to be captured onto the electrode surfaces and to form spherical cell-aggregates between the electrodes by applying p-DEP and n-DEP, respectively (Figure 1D). Figure 1E shows the simulation result of electric field strength on the saw-shaped and line-shaped indium tin oxide (ITO) electrodes by FEM. Compared to the line-shaped electrodes, the saw-shaped electrodes have the advantage of efficient cell enrichment. As shown in Figure 1E, the tips of the saw-shaped electrodes showed higher electric field strength than those of the line-shaped electrodes with the same applied voltage. Thus, a small amount of rare and precious cells can be effectively and rapidly trapped on the tips of the electrodes by strong p-DEP with low applied voltage.

**Figure 2:**
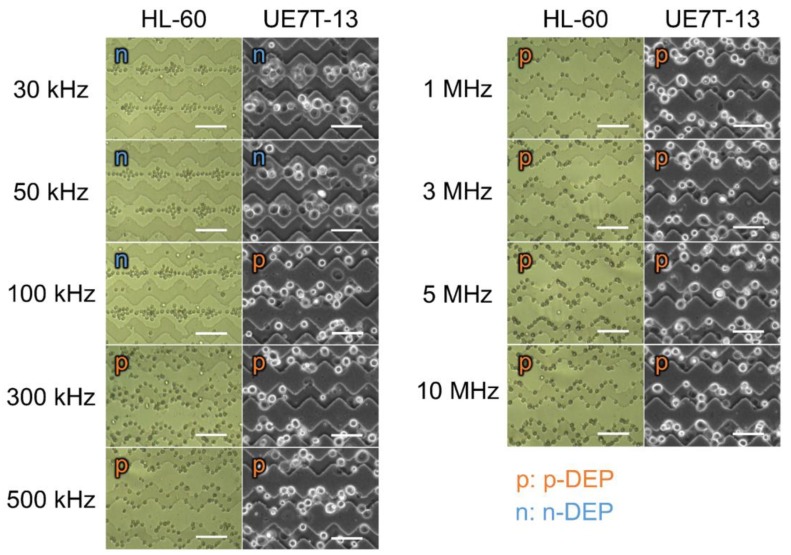
Microscopic images of cell dielectric responses at various frequencies. DEP behaviors of the HL-60 and UE7T-13 cells on the saw-shaped electrodes at 20 V peak-to-peak (Vpp) at various frequencies. The concentrations of the UE7T-13 and HL-60 cells were 1.0 × 10^7^ cells/mL, and the images of these cells were obtained by bright field and phase contrast microscopy, respectively. Scale bars represent 100 μm.

Before cell separation, the DEP behaviors of the UE7T-13 and HL-60 cells were observed under an optical microscope under conditions ranging from 30 kHz to 10 MHz (Figure 2). The concentrations of the UE7T-13 and HL-60 cells were 1.0 × 10^7^ cells/mL. The UE7T-13 cells experienced n-DEP at frequencies of 30 to 50 kHz and p-DEP from a frequency of 100 kHz onwards (see Supplementary movies, S1 and S2). In case of the HL-60 cells, n-DEP was observed at frequencies of 30 kHz to 100 kHz and their p-DEP was observed from a frequency of 300 kHz onwards. These results show that the DEP behaviors of the UE7T-13 and HL-60 cells were different at 100 kHz frequency, suggesting that this DEP device can separate heterogeneous cells derived from bone marrow without any labeling.

### 3.2. Cell Viability Assay after DEP

The viability of the UE7T-13 cells was investigated by live/dead assay using the Live-Dead Cell Staining Kit (BioVision Inc., Milpitas, CA, USA) after application of p-DEP for 5 min and culture for 2 h. After DEP treatment, the sucrose solution was replaced by culture medium. The UE7T-13 cells were incubated at 37 °C and 5% CO_2_. The result showed that the UE7T-13 cells spread normally, and their viability was 90.1 ± 3.5%.

### 3.3. Cell Separation and BMSC Enrichment

**Figure 3:**
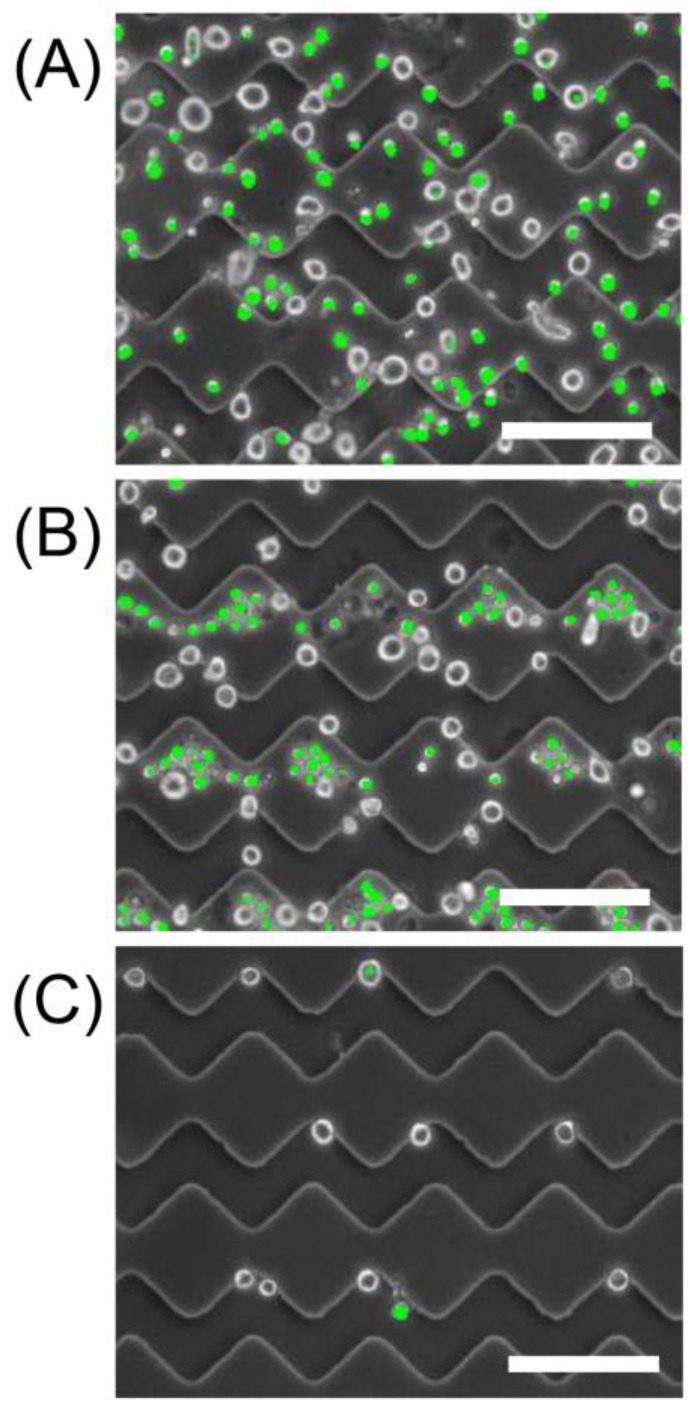
Phase contrast images during bone marrow-derived mesenchymal stem cells (BMSCs) separation and enrichment. The UE7T-13 cells and HL-60 cell suspensions were injected into the DEP device (**A**). The UE7T-13 cells were separated on the saw-shaped ITO electrodes after application of DEP with 5 Vpp at 30 kHz (**B**), and the HL-60 cells were washed out selectively under DEP (**C**). The HL-60 cells were stained with fluorescent reagent Live-Dye to identify the UE7T-13 cells. The concentrations of the UE7T-13 and HL-60 cells were 1.0 × 10^7^ cells/mL and 2.0 × 10^7^ cells/mL, respectively. The images were merged by binarized fluorescence images at the same field. Scale bars are 100 μm.

To confirm the separation ability of the constructed device, a mixture of UE7T-13 and HL-60 cells was injected into the device. Figure 3 shows the microscopic image of cell separation and enrichment. The size of a UE7T-13 cell is larger than that of a HL-60 cell, and the concentrations of UE7T-13 and HL-60 cells were 1.0 × 10^7^ cells/mL and 2.0 × 10^7^ cells/mL, respectively. Only the HL-60 cells were stained with the fluorescent reagent Live-Dye (BioVision Inc., Milpitas, CA, USA) in order to identify the UE7T-13 cells before mixing the cells (Figure 3A). After optimization of the experimental conditions for AC voltage and frequency, the UE7T-13 cells were captured on the electrodes, while the HL-60 cells were repelled from the electrodes (Figure 3B). Moreover, most of the HL-60 cells were removed after washing with sucrose solution under DEP (Figure 3C). Although the same buffer and 30 kHz of frequency were used in Figure 2 and Figure 3, the DEP behaviors of the UE7T-13 cells in both experiments were opposite. This suggests that the differences in total cell concentrations and the scale of AC electro-kinetic flow generated by high voltage might affect the DEP behaviors of the UE7T-13 cells [25,26]. The purity, recovery, and enrichment rate of the UE7T-13 cells after DEP separation were 83.5 ± 7.1%, 29.1 ± 4.1%, and 2.3, respectively. The ratio of cells lost by washing process was about 70%. Since separation by the DEP device is completed within 5 min, the total enrichment procedure, including replacement of solution, DEP separation, and washing, can be completed within 40 min only. Therefore, these results show that extremely rapid separation and enrichment of BMSCs from a heterogeneous cell mixture with promyelocytes, which was also derived from bone marrow, was successfully achieved using the DEP device with the saw-shaped electrodes. After further optimization of the DEP condition by changing frequency and voltage, the recovery rate and enrichment value could be improved. Additionally, the flow rate of washing solution also improves the recovery rate and enrichment value. In the present study, since washing solution were manually injected using micropipettes, the flow rate could not be controlled and fast flow rate was generated, which causes a decrease in the recovery rate and enrichment value of BMSCs. Construction of a microfluidics device possessing our saw-shaped DEP electrode, which is connected to a syringe pump for generating constant slow flow rate, would show high performance for BMSC isolation and enrichment. The real bone marrow sample includes many cell species such as hematopoietic cells, marrow adipose tissue, and supportive stromal cells. Thus, the target BMSCs may not be obtained by single washing operation, though repeated washing operation under different optimized separation conditions would isolate BMSCs from real samples.

## 4. Conclusions

In summary, a DEP device with saw-shaped electrodes was fabricated for the separation and enrichment of BMSCs, using a mixture of UE7T-13 and HL-60 cells. Compared to line-shaped electrodes, the saw-shaped electrodes have the advantage of efficient cell enrichment owing to the generation of higher electric field gradient with the same applied voltage. After optimizing the frequency of the applied voltage, bone-marrow-derived MSCs were rapidly and non-invasively separated and enriched from promyelocytes derived from bone marrow, without any labelling. This is the first study to show label-free rapid separation and enrichment of BMSCs within 5 min from a heterogeneous cell mixture derived from bone marrow. Our results suggest that cell separation and isolation by DEP with saw-shaped electrodes could be an important technique in the field of stem cell research and therapy.

## Supplementary Materials

The Supplementary Materials are available online at http://www.mdpi.com/1424-8220/18/9/3007/s1, Movie S1: n-DEP of UE7T-13 at 20 Vpp with 30 kHz, Movie S2: p-DEP of UE7T-13 at 20 Vpp with 1 MHz.
